# Patient‐derived organoids, creating a new window of opportunities for pancreatic cancer patients

**DOI:** 10.15252/emmm.202215707

**Published:** 2022-03-14

**Authors:** Sandhya Sandhya, Tara L Hogenson, Martin E Fernandez‐Zapico

**Affiliations:** ^1^ Schulze Center for Novel Therapeutics Division of Oncology Research Mayo Clinic Rochester MN USA

**Keywords:** Cancer, Digestive System

## Abstract

Standard‐of‐care regimens for pancreatic ductal adenocarcinoma (PDAC) include a combination of chemotherapies, which are associated with toxicity and eventually tumor resistance. The lack of relevant tool to identify and evaluate new therapies in PDAC necessitates the search for a model, especially for cases with treatment resistance to standard of care. In the study from Peschke *et al* (2022), they describe a longitudinal platform to identify drug‐induced vulnerabilities following standard‐of‐care chemotherapy treatment using patient‐derived organoids (PDOs) providing an opportunity to predict therapeutic response and define new treatment vulnerability induced by standard of care. Previously, tumor resistance to chemotherapy has typically been described as selection for resistant tumor cell populations. However, Peschke *et al* (2022) demonstrated that PDAC cells seemed to acquire resistance not only through genetic changes, but also through modifications in cellular plasticity leading to gene expression and metabolism changes. Thus, the study supports this type of platform for the identification of new therapeutic targets following standard‐of‐care treatments in PDAC.

## Graphical abstract

M. E. Fernandez‐Zapico, T. L. Horgenson, and S. Sandhya discuss advantages of a longitudinal platform using patient‐derived organoids to identify new treatment regimens for pancreatic ductal adenocarcinoma patients following standard‐of‐care chemotherapy treatment as reported by M. Reichert and colleagues, in this issue of EMBO Mol Med.

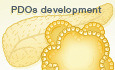

Pancreatic ductal adenocarcinoma (PDAC) is a highly malignant cancer of pancreas with a 5‐year survival rate of ~10%. Early metastatic progression, highly resistance to all current standard‐of‐care therapies including radio‐, and chemotherapy, makes it a devastating disease (Orth *et al*, 2019). Surgical resection along with chemotherapy has curative potential but is only applicable to ~20% of cases since the majority of patients are diagnosed with advanced disease and are not candidate for surgical resection. Commonly used regimens for PDAC treatment include gemcitabine (Gem) + nab‐paclitaxel or FOLFIRINOX (5‐fluorouracil, leucovorin, irinotecan, and oxaliplatin). Though between‐trial comparisons are not statistically valid, the perceived efficacy of FOLFIRINOX makes it the preferred first‐line treatment option in fit patients. Additionally, Gem‐based regimens are reserved for less fit or elderly patients due to superior tolerability. These chemotherapy regimens are associated with inevitable toxicity and resistance and only increase the median survival for approximately a year. Thus, if individualized chemotherapy sensitivity in relevant models can be predicted, anticipated efficacy could be enhanced while minimizing toxicity.

Cell lines, mice xenograft, and genetically engineered mice are the widely used models to evaluate therapeutic responses, but they do not closely represent the genetic and epigenetic landscape of each PDAC patient. Tumor tissues derived from patient are considered most representative but are associated with limitations such as availability and low amount. Apart from these, tumors are good for molecular profiling but are not suitable for pharmacological testing, which is the current requirement for PDAC treatment where patients show very high variability to drug response. To circumvent the limitations of the above models, patient‐derived organoids (PDOs) provide advantages by being evolved as a reliable model to predict the drug response in cells having characteristics of individual patient tumors. Also, these 3‐dimensional (3D) cultures have heterogeneous structures mimicking the *in vivo* tumor architecture. Studies show PDO responses to drug correlate well with the genomic profile of the patient tumor with a high sensitivity and specificity (Grossman *et al*, 2022).

In the current issue, Peschke *et al* (2022) describe the development of a longitudinal precision oncology platform to gain insights into chemotherapy‐induced vulnerabilities, to improve treatment response in the clinical setting. Using generated PDOs derived from biopsy and resection samples of a patient before and after FOLFIRINOX treatment, the authors found PDOs show re‐differentiation post‐FOLFIRINOX treatment. They also found there was a treatment‐induced metabolic switch from aerobic glycolysis to oxidative phosphorylation and increased lipid metabolism in the resistant PDO. In addition, RNA‐seq analysis of these pre‐ and post‐chemotherapy PDOs found similar classical subtype for both the models. In fact, characteristic pathways of basal‐like subtype were more enriched in PDO before chemotherapy treatment. The molecular switching of pathways from basal‐like subtype to classical following FOLFIRINOX therapy may provide a proliferate advantage and possibly in the development of resent phenotype (Chan‐Seng‐Yue *et al*, 2020; Grünwald *et al*, 2021). Importantly, this study showed the adaptive response of the tumor to chemotherapy through robust proliferation to escape/resist the treatment without significantly changing the genetic landscape (Fig 1).

**Figure 1:**
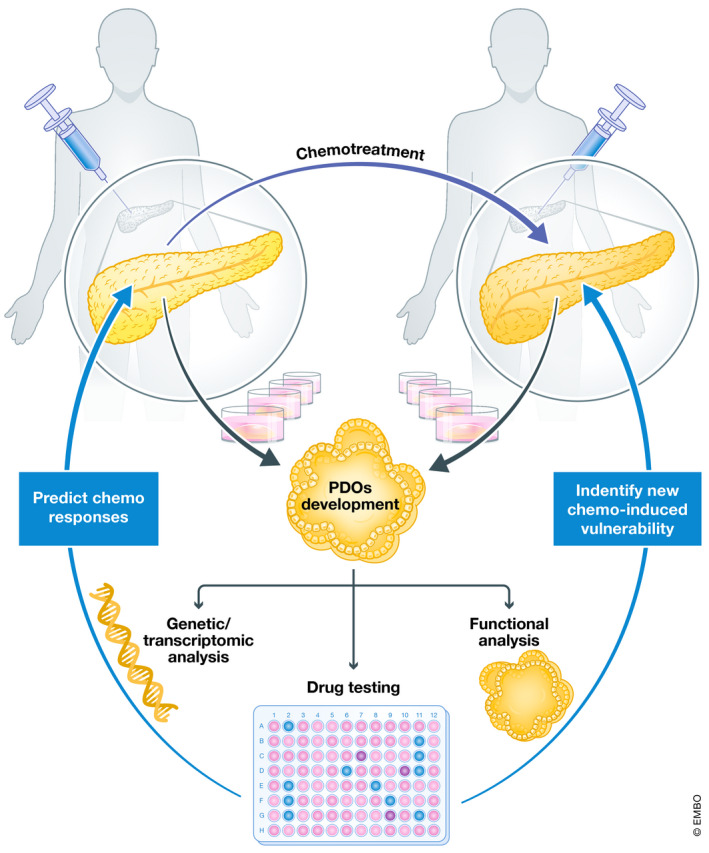
PDOs, a model to predict the patient response to chemotherapy and define new treatment‐induced vulnerability Treatment‐induced plasticity is the major cause of treatment failure in PDAC. PDOs derived before and after treatment can be used to predict the drug response as well as to identify chemo‐induced vulnerability and provide an opportunity to select effective treatment regimen for patient. (Image generated on biorender.com).

Further analysis identified the FOLFIRINOX treatment generated a resistance phenotype by inducing changes in cellular plasticity leading to new drug sensitivities. Peschke *et al* (2022) performed unbiased drug screen for 415 compounds and narrowed down to 3% of the tested drugs having differential responses between the sensitive and resistant PDOs. Post‐treatment PDOs were resistant to Aurora kinase inhibitor, eIF4E/eIF4G interaction inhibitor, and SMAC mimetics. They were sensitive to inhibitors of the ATPase p97/valosin‐containing protein (CB‐5083 and NMS‐873), the epidermal growth factor receptor (lapatinib and poziotinib), and MEK (trametinib, cobimetinib, and BI‐847325). Since the KRAS‐MEK‐ERK axis is a major driver of PDAC tumorigenesis, Peschke *et al* (2022) validated MEK inhibitor (MEKi) sensitivity in a larger cohort of patients and confirmed chemotherapy‐induced vulnerability of cells following neoadjuvant therapy. Using PDOs, this study identified treatment‐imposed cell plasticity with diverse response for polychemotherapy as well as relevant targeted therapies.

The authors demonstrate a vulnerability of cancer cells following chemotherapy treatment that is independent of genetic mechanisms. This demonstrates a need for additional testing using PDOs to identify new treatment regimens for sensitivity that evaluate more than the genetic landscape of the tumor. Additional studies examining these new pathways altered following chemotherapy treatment could benefit PDAC treatment options. The PDO model used in the study makes it a highly reliable and specific model for studying these mechanisms. This demonstrates another potential use of PDOs for identifying new cancer therapies for PDAC patients.
